# The many questions on the use of biomarkers for neurodegenerative diseases in clinical practice

**DOI:** 10.3389/fnagi.2014.00045

**Published:** 2014-03-18

**Authors:** Manuel Menéndez-González

**Affiliations:** ^1^Neurología, Hospital Álvarez-BuyllaMieres, Spain; ^2^Morfología y Biología Celular, Universidad de OviedoOviedo, Spain; ^3^Instituto de NeurocienciasOviedo, Spain

**Keywords:** biomarker, dementia, clinical practice, mild cognitive impairment, treatment outcome

There are two basic questions always raised when biomarkers are proposed to be used for diagnosing neurodegenerative diseases (ND) in clinical practice (Wilner, 2010). The first question is, do biomarkers enhance the diagnostic accuracy? Clinical-based diagnosis accuracy of ND varies depending on the disease, stage and the criteria used, but in general terms it is about 75–90% accuracy. Today, there is consensus that several biomarkers, combined with the traditional clinical process, may allow a more accurate diagnosis in many ND (Galluzzi et al., 2013). This fact is particularly important in early stages, when the diagnosis is specially challenging. For most patients with mild deficits concerned about the development of ND, a careful history, and a physical, neurological and neuropsychological evaluation with a close follow-up (a “wait and see” approach) used to be the standard practice. Today we can offer a more proactive approach for discerning whether there is an underlying neurodegenerative process behind these mild deficits (Heister et al., 2011). The National Institute on Aging-Alzheimer's Association criteria for AD or MCI recommend the use of amyloid ligands with caution, and only in exceptional circumstances they should be used in clinical practice (Albert et al., 2011; Sperling et al., 2011). The use of different biomarkers for differential diagnosis should not be discouraged. On the other hand, use of biomarkers as predictive instruments should be discouraged.

The second question is, does the additional diagnostic accuracy provided by biomarkers really matter? In clinical practice, a test that confirmed or ruled out a ND would remove uncertainty. This would also disregard or reinforce the need to consider other diseases that may present symptoms similar to those of ND. In some of these diseases, prompt diagnosis can lead to earlier effective treatment, such as shunting for normal pressure hydrocephalus, supplementation with thyroid hormones in hypothyroidism or antidepressive medications for depression.

The best argument for using biomarkers in clinical practice would be the possibility of treating patients with a disease-modifying therapy that prevents or delays the progression of the disease. In other words, putting patients on drugs with neuroprotective effect. However, no drug have proven prevention of any ND yet. The current therapies only provide symptomatic improvement at best so there is an urgent need to discover neuroprotective treatments. But how can we conduct clinical trials for testing such drugs when diagnosing ND at its very early stages is so difficult? Again, the support of biomarkers should be mandatory for enrolling patients in research studies.

Then, if there is not a disease-modifying therapy yet, what is the importance of an early diagnosis in routine clinical practice today? There are several reasons to make an early diagnosis even when we cannot modify the course of the disease. For instance, once people become demented, they can no longer plan for their future or dictate their end-of-life care. An early diagnosis of Alzheimer's disease gives a person the opportunity to decide on important questions before he or she gets demented (Martínez-Rivera et al., 2008). It also has important consequences for the patient's family. However, an early diagnosis of a ND may also have negative psychological consequences in an otherwise well-functioning person who must now consider an inexorable decline towards a state of illness and dependency. Consequently, the pros and cons of early diagnosis must be carefully weighed up in each individual prior to perform a confirmatory test.

And even if we have decided to use biomarkers for supporting the diagnosis of a ND, there are many questions to face. It is important to emphasize that standardization of these biomarkers is currently limited, and results often vary from laboratory to laboratory. Ultimately, it will be necessary to interpret biomarker data in the context of well-established normative values. Moreover, procedures for acquisition and analysis of samples need to be established to implement these biomarker criteria on a broad scale. Although we consider biomarkers as “negative” or “positive” for purposes of classification, it is recognized that varying severities of an abnormality may confer different likelihoods or prognoses, which is difficult to quantify accurately for broad application. Currently it is difficult to understand the relative importance of different biomarkers when used together, and to interpret results when biomarker data conflict with one another.

Equally important, there is a dearth of truly predictive studies at the individual subject level or in unselected populations. The use of biomarkers in the clinical practice will require the ability to assign a likelihood of progression in an individual person over a specific time interval through the use of a single or multiple biomarkers. Another major limitation is knowledge about the timing of decline because the ability to detect change is dependent on the period of observation or prediction. A complete understanding of the role of biomarkers in prediction of decline will require both short and long-term periods of observation.

Finally, little is known about outcome when biomarkers provide conflicting results. When a panel of biomarkers is used, it is possible that for some individuals, one biomarker will be positive, one will be negative, and one equivocal. The long-term significance of such findings may also vary with the length of follow-up.

Therefore, questions such as “what biomarker is better for making the early diagnosis of each ND?,” or “which one is better for the follow up,” “which one for making the differential diagnosis with other disease?”, “how to interpret the results of these tests in coordination with clinical or genetic findings” and “how to combine the results from different biomarkers?” have important repercussion on the management of patients suspected of suffering from ND and still remain unresponsed. The answers to these questions are not always easy and rely on upcoming science.

We need more data and more networking to find appropriate conclusions. Collaboration between basic, translational and clinic researchers is paramount for giving answers relevant to everyday clinical practice. In this regard, the topic research issue accompanying this editorial letter gathers together a bunch of review and original articles exploring the use of biomarkers for ND from different perspectives.
